# Measuring the geographic disparity of comorbidity in commercially insured individuals compared to the distribution of physicians in South Africa

**DOI:** 10.1186/s12875-022-01899-1

**Published:** 2022-11-17

**Authors:** Cristina Mannie, Stefan Strydom, Hadi Kharrazi

**Affiliations:** 1 Johns Hopkins Bloomberg School of Public Health, 624 N Broadway, Office 606, Baltimore, MD 21205 USA; 2 Mast Analytics Pty (Ltd), 4th Floor Sunclare Building, 21 Dreyer St, Claremont, Cape Town, 7708 South Africa; 3 Johns Hopkins School of Medicine, 2024 E Monument St, Baltimore, MD 21205 USA

**Keywords:** Health equity, Healthcare disparity, Comorbidity index, Geographic analysis, Insurance claims, South Africa

## Abstract

**Background:**

Measuring and addressing the disparity between access to healthcare resources and underlying health needs of populations is a prominent focus in health policy development. More recently, the fair distribution of healthcare resources among population subgroups have become an important indication of health inequities. Single disease outcomes are commonly used for healthcare resource allocations; however, leveraging population-level comorbidity measures for health disparity research has been limited. This study compares the geographical distribution of comorbidity and associated healthcare utilization among commercially insured individuals in South Africa (SA) relative to the distribution of physicians.

**Methods:**

A retrospective, cross-sectional analysis was performed comparing the geographical distribution of comorbidity and physicians for 2.6 million commercially insured individuals over 2016–2017, stratified by geographical districts and population groups in SA. We applied the Johns Hopkins ACG® System across the claims data of a large health plan administrator to measure a comorbidity risk score for each individual. By aggregating individual scores, we determined the average healthcare resource need of individuals per district, known as the comorbidity index (CMI), to describe the disease burden per district. Linear regression models were constructed to test the relationship between CMI, age, gender, population group, and population density against physician density.

**Results:**

Our results showed a tendency for physicians to practice in geographic areas with more insurance enrollees and not necessarily where disease burden may be highest. This was confirmed by a negative relationship between physician density and CMI for the overall population and for three of the four major population groups. Among the population groups, the Black African population had, on average, access to fewer physicians per capita than other population groups, before and after adjusting for confounding factors.

**Conclusion:**

CMI is a novel measure for healthcare disparities research that considers both acute and chronic conditions contributing to current and future healthcare costs. Our study linked and compared the population-level geographical distribution of CMI to the distribution of physicians using routinely collected data. Our results could provide vital information towards the more equitable distribution of healthcare providers across population groups in SA, and to meet the healthcare needs of disadvantaged communities.

**Supplementary Information:**

The online version contains supplementary material available at 10.1186/s12875-022-01899-1.

## Background

Disparities in equitable healthcare resources is a global challenge [1–3]. Many countries are experiencing an increasing rate of disparities in providing impartial healthcare resources to disadvantaged populations and underserved communities [1, 4]. Several attributes, including race, ethnicity, socioeconomic status, gender, and geographic location, have caused communities and populations to systematically experience greater obstacles to accessing equal healthcare resources [3, 5].

Prior research on measuring disparities in receiving healthcare resources has often focused on individual diseases [6]. Individual diseases provide useful evidence on disparate outcomes caused by or correlated with various social determinants of health [7]. However, such studies do not reveal the negative effect of individual diseases when compounded together as comorbidities. Incorporating comorbidities in disparities research provides a useful approach to assessing the total burden of disease in underprivileged populations [6]. Nonetheless, measuring comorbidities in a population is challenging and requires calculating validated comorbidity indices [8, 9] across a large number of individuals using data collected across health providers [10, 11].

Morbidity studies in South Africa (SA) have traditionally focused on individual diseases [12] or a combination of few, select, commonly occurring chronic diseases [13–15], have been based on small and often geographically limited samples [15], and have involved time-consuming, non-routine data collection processes [12, 14]. To address these limitations, in a past study, we leveraged insurance claims data and used a validated comorbidity index to measure the total burden of disease across 2.6 million commercially insured individuals residing in different SA geographies [10]. The study served to highlight how a measure of comorbidity can be used to provide a more in-depth understanding of the current and future healthcare needs through its determination of individual-level health status. Additionally, the aggregated comorbidity data described the impact of geographic regions on potential health inequities. The study concluded that comorbidity indexes should be further investigated as a means to detect geographical areas of increased and potential unmet healthcare needs [10].

To further assess the effect of geographic and racial disparities in accessing impartial health resources, this research measures the unequal distribution of comorbidities in SA compared to healthcare providers associated with the study population. Results of this study provide a novel perspective on health inequalities in SA, which could be used to support decision-making related to the equitable allocation and distribution of limited healthcare resources based on total burden of diseases.

The objectives of this study are to: (1) present the measurement of comorbidities as an underutilized perspective when quantifying disease burden and planning resource allocation; (2) present the use of a comorbidity measure/index as a novel means to identifying health equities by considering acute and chronic diseases co-existing at an individual level as a better perspective of total burden facilitating the identification of inter-individual and between-group differences in outcomes; (3) showcase how linking comorbidity patterns in relation to current healthcare resources can serve the assessment of the availability and distribution of resources required to address (unmet) healthcare needs to improve population health; and (4) to show how routinely collected health data could be used to monitor co-morbidities nationally more easily, timeously and cost-effectively than existing periodic morbidity studies, while also providing a practical means of identifying potentially underserved areas (to be used in conjunction with more robust studies) to improve health services delivery.

## Methods

### Measure of health status

We used the validated Johns Hopkins Adjusted Clinical Grouper (ACG®) risk score as the measure of health status [9, 16]. The ACG® risk score is a patient-centered summary measure based on the premise that sicker individuals need more healthcare resources to adequately manage their health. The ACG® System classifies the morbidity level of each individual by considering the age, gender and particular pattern of morbidity (i.e., acute and chronic conditions) experienced by the individual over the past twelve months [8]. Based on this information, each individual is assigned to one of 105 mutually exclusive ACG® risk groups. Individuals within the same ACG® risk group have morbidity levels requiring similar needs for healthcare resources. A risk score/weight is then assigned to each risk group based on the average annual healthcare resource utilization of the individuals in the group representing the average cost expected to manage the particular combination of clinical conditions experienced by individuals [9]. At an individual level, this risk score describes the relative healthcare resources need for the individual’s morbidity level compared to the average individual in the population. The ACG® System has been shown to explain significantly more variation in utilization than demographics-only models [17–19].

A set of age-related risk scores were determined based on the average annual healthcare resource utilization by age. Age-adjusted ACG risk scores were calculated by dividing each individual’s risk score by the age-only risk weight.

By aggregating the individual risk scores of individuals of interest, such as in a specific geographical area (e.g., district) or of a subpopulation, the average risk for the group of individuals described as a comorbidity index (CMI) can be determined [9]. This CMI describes the average healthcare resource needs of the group of individuals based on their combined morbidity and can thus be used to compare the degree of morbidity and expected healthcare utilization of one population to another. By calibrating the CMI of the entire study population to equal 1, the CMI of each geographical region can be compared relative to the CMI for the entire population highlighting the different levels of morbidity and resulting expected healthcare needs for different regions. CMIs above or below a value of 1 are expected to experience higher or lower healthcare utilization (and implied higher or lower morbidity) compared to the average morbidity level and the average cost per life observed for the entire population.

### Study design

A retrospective, cross-sectional analysis was performed comparing the ACG® CMI scores for commercially insured individuals living in the same area between 2016 and 2017 in relation to the geographical distribution of general practitioners (GPs) and specialists (SPs) from whom healthcare services were claimed for over the same two-year period. GP and SP density were calculated by considering the number of individuals, GPs, and SPs within a 5 km radius of each individual’s residential location. The 5 km radius is a national standard considered to represent reasonable access to primary healthcare services, and is based on a maximum walking time of one hour at a normal pace (approximately 4.5 km/hour) [20, 21]. The location of each individual was determined using the smallest geographical unit in our study known as the electoral ward and taken as the center of the respective ward. By considering access to GPs and SPs within a defined radius, we were able to include physicians who technically practice outside of the ward but within a distance considered reasonable proximity for primary health care services [20, 21].

### Data sources

Administrative claims data were obtained from one of the largest health risk management services providers and administrators of health plans in SA. Data used in this study included: de-identified, aggregated patient-level data for 2016 and 2017 of total claims costs; the ACG® risk score which provides a measure of the clinical and financial risk of each member for each year; claiming healthcare services providers by specialty for 2016 and 2017; basic demographic data (i.e., age, sex, population group); and, geographical region for individuals and healthcare providers as determined by postal codes recorded on health plan administration records.

**Fig. 1:**
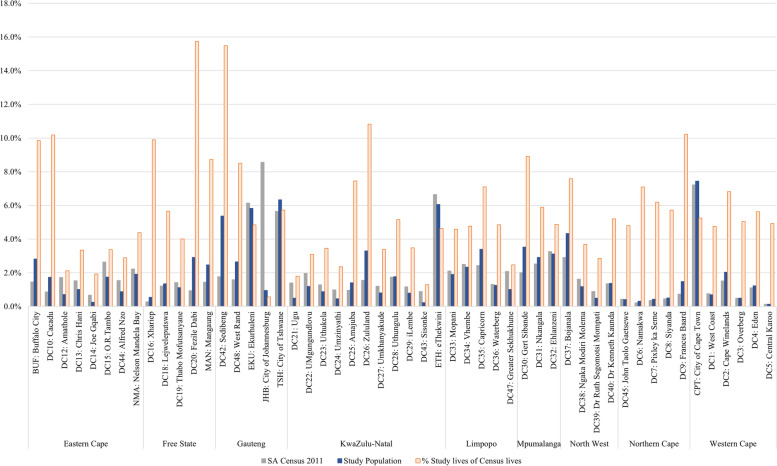
A comparison of the distribution of commercially insured individuals represented in this study with the distribution of 2011 Census populations per district. The grey and blue bars show the proportion of census lives and study lives per district respectively. The peach bars show the number of study lives as a percentage of census lives per district. Higher bars indicate over-representation and lower bars indicate under-representation in the study sample relative to the national population. (Source: Authors’ work)

The population groups in this study are described using the four major racial groups used by SA’s Census to classify the population [22]. Characteristics of the study population were compared to the 2011 SA Census data (extracted from Statistics South Africa’s online database [22]) to measure the representation of the national population by the study denominator (Fig. 1; see Additional file 1).

Postal codes recorded for health plan members and healthcare providers were linked to the 2016 demarcated boundaries for electoral wards and then mapped using SA Census data to the respective districts. The maps of SA were produced in Python using geographical shapefiles for districts in SA obtained from publicly available sources as published by the Municipal Demarcation Board [23]. The mapping of postal codes to electoral wards and then to districts was made possible by an allocation method that assigns each postal code to a mutually exclusive electoral ward. Coordinates for postal codes used to determine the proximity of individuals to healthcare providers were extracted from the GeoNames database [24].

### Study population

The population assessed for inclusion in this study included 3.96 million members enrolled in SA health plans in 2016 and 2017 for whom comprehensive managed care services were provided by the administrator. The study population consisted of members from open health plans (i.e., plans that anyone can join) and closed health plans (i.e., restricted to employees of organizations). All health plans are obligated by legislation to cover a standard set of benefits—Prescribed Minimum Benefits (PMBs)—and are subject to conditions of open enrolment and community rating [25, 26].

The final study population of 2.64 million individuals was determined using two main criteria. The first criterion restricted health plan members to those with a minimum membership of at least 6 months in each year to ensure sufficient time over which to collect clinical diagnosis information for the ACG® System to reasonably assign the member to an actuarial risk group representative of the member’s clinical and financial risk in a given year. The 6-month minimum membership requirement for the assignment of ACG® risk scores is commonly recommended to counter newly enrolled individuals with recent healthcare needs which may overstate healthcare costs over shorter periods [9]. Due to almost complete membership for the majority of the study population in each year, healthcare costs (and thus risk scores) per individual were deemed representative of annual healthcare costs.

The second criterion included health plan members for whom a single ward (i.e., geographical location) over the two years was identified so as to link the health status of individuals during the entire study period to a single region to which the individual was exposed for an extended period likely to have impacted their concurrent health status. By ensuring the same geographical region in both years, confounding factors expected to influence healthcare utilization and the ACG® risk score were assumed to be mainly constant. Since health status is dynamic and expected to change from time to time, the average morbidity and average healthcare costs of individuals with membership across both years were used to provide a better indication of the health status and healthcare resource needs of each individual as opposed to using only a single year. Details of the inclusion criteria applied in this study are published elsewhere [10].

A geo-level power and sample size analysis was performed as part of a prior study on this population and dataset to ensure that the geographical districts included in this study have adequate individuals to represent the underlying commercially insured populations in each district. The sample size also ensured that notable differences in CMI (or average healthcare utilization) could be detected when compared to the national average of at least a 5% difference [10].

### Statistical analysis

The database management and analysis for this study were performed using MySQL (v5.7.22). Statistical analysis and geographical plots were produced using R (v3.4.3) and Python (v3.7.8). The R package pwr (v1.2–2) was used for the power analysis. Python packages used for the statistical analysis and geographical plots produced included GeoPandas (v0.8.1), Matplotlib (v3.3.2) and statsmodels (v0.12.2). Graphs were created in Microsoft Excel 365 (2020).

Within the ACG® System, the ACG® risk groups are used to directly explain variation in total cost and provide an easily calibrated model for explaining the relative risk [16]. The risk weights for each group were derived by taking the mean healthcare expenditure for all individuals in a risk group divided by the mean healthcare expenditure of all individuals in the population (indirect standardization) such that the average cost weight across the population is equal to one to facilitate comparison. The risk weights calculated based on the ACG System’s reference U.S. population were highly correlated with the weights empirically determined using local data. Despite this finding, weights were recalibrated using local SA cost data reflective of local healthcare benefits and utilization experience for the study population. Further details have been published in a prior study based on the same population [9, 10].

Two linear regression models were constructed to test the relationship between ACG® risk scores, age, gender, population group and population density against GP and SP density, respectively. Continuous variables were scaled between 0 and 1 to facilitate the interpretation of model coefficients on the same scale. Additional information regarding the choice of linear regression as the main statistical method is provided (see Additional file 3). GP and SP density was defined based on a 5 km radius between study individuals and providers as defined earlier. The regression analysis was repeated using a 10 km radius instead of a 5 km radius to test the sensitivity of this choice; results were found to be consistent with our main findings (see Additional file 3).

This study involved secondary data use and was approved by the Institutional Review Board of the Johns Hopkins Bloomberg School of Public Health, Baltimore, Maryland.

## Results

### Characteristics of the study population

The study population of 2.64 million commercially insured individuals represented approximately 5% of the national population and 34% of all commercially insured individuals in SA (See Additional file 1) [25]. In comparison to the national distribution (reported by the SA Census 2011), the study population has comparable proportions of individuals by gender, across all ages, and provinces. Differences in the study population compared to the national population, however, included a higher proportion of White members and a lower proportion of Black African members, and lower proportions of individuals between the age of 20–30 years resulting in a potential bias towards older individuals (i.e., average age 31.6; SA 2017 median age 26.6) with existing healthcare needs and expected higher utilization. Unlike the relatively high unemployment rate experienced nationally by approximately a third of the population [27], the study population is typically formally employed [25]. Additional comparison of the study population versus SA population has been conducted in prior research [10].

Conventional statistical testing was not applicable for comparing significant differences between the distribution of lives across the study population and the 2011 Census, due to the large samples in each subgroup making even small differences in proportions appearing to be significant [28, 29].

SA’s 9 provinces contain 52 districts which are divided into 4277 smaller geographical units known as electoral wards [22]. The study population represented a third of all wards (*n* = 1427) and all 52 districts.

### Geographical distribution of study lives versus 2011 census lives

We compared the geographical distribution of the analyzed study population (blue bars) to the distribution of the 2011 Census lives (grey bars) by district in SA (Fig. 1; see Additional file 1). The study population was concentrated in the City of Cape Town in the Western Cape (along the West coast), the City of Tshwane in Gauteng (North) and Ethekwini in KwaZulu-Natal (East coast); three of the five most populous districts in SA. In addition, we calculated the proportion of individuals (peach bars) represented by the study population for each district relative to the 2011 Census lives (with the assumption that individuals in both periods were the same) as an approximate indication of districts in which greater or poorer representation may exist. Figure 1 shows that the City of Johannesburg, which is the most populous district in SA, was underrepresented in this study, making up 1% of the study population compared to 8.6% of the national population in 2011.

### Geographical distribution of CMI

**Fig. 2:**
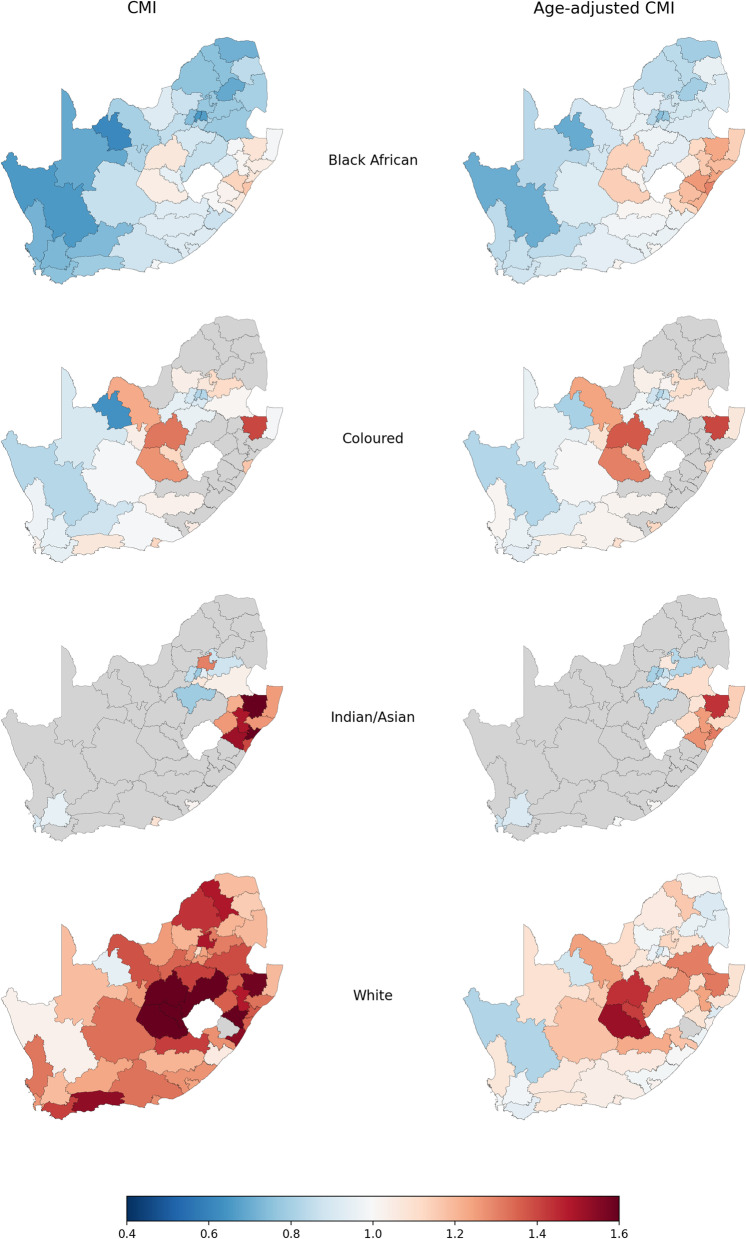
A comparison of average morbidity per district and population group measured by the ACG CMI before (left) and after (right) age-standardization. (Source: Authors’ work)

A comparison of the geographical distribution of comorbidity amongst the study population by district was performed (Fig. 2). To better understand the impact on CMI by other factors such as increased age for whom increased healthcare costs are expected, we compared changes in CMI before and after accounting for the influence of age on the individuals in each district (Fig. 2 left vs. right). We also analyzed the influence of population group (available in our study data) and compared the CMI per district stratified by each population group (Fig. 2 rows).

The CMI for a district is calculated as the average ACG risk score of all study individuals residing in the district. At the district level, the CMI is a measure of the expected healthcare utilization in the district relative to the overall healthcare utilization in the study population. The CMI is expressed as a relative measure, with a value of 1.0 indicating the expected utilization is equal to that of the overall study population. Districts in the highest CMI range of 1.4 to 1.6 are expected to experience 40% to 60% higher healthcare utilization than the overall study population based on the known demographic and clinical risk factors of individuals in the district. Conversely, districts with CMI scores of less than 1.0 are expected to incur lower levels of healthcare utilization relative to the overall study population.

Figure 2 highlights the districts in which the highest healthcare utilization is expected based on disease burden as represented by the CMI value. Overall, districts with higher levels of disease burden (i.e., CMI values represented by shades of red) compared to other parts of the country appeared concentrated in the Western Cape, Free State and KwaZulu-Natal provinces. In contrast, the Eastern Cape, and especially the Northern Cape and Limpopo provinces, appeared to have the lowest CMI scores (i.e., shades of blue) in the country (Fig. 2). A labelled map of districts in SA is provided as reference (see Additional file 2).

To investigate whether districts with high CMI scores were mainly driven by the age of the population, we calculated an adjusted CMI score in which the impact of age was excluded. Figure 2 (right column) shows that, for the most part, similar districts have relatively high and low CMI values respectively in the age-adjusted analysis, although the difference between districts is generally reduced. Districts with much lower CMI values after age adjustment – notably Overberg and Eden along the South-West coast in the Western Cape province – indicate a relatively high proportion of older adults that contributed to their risk scores. Conversely, districts with much higher CMI values after age adjustment – notably the Capricorn district in the Limpopo province in the North of the country – indicate a relatively young population.

The CMI associated with Black African individuals in the study population stands out from the other population groups (i.e., most geographies with shades of blue in the first row of Fig. 2), indicating lower than average healthcare utilization compared to other population groups. In contrast, the geographical plots of White and Indian/Asian individuals indicate several districts expecting to incur higher than average healthcare utilization relative to other population groups (Fig. 2, last two rows).

After adjusting for age, our findings identified a cluster of districts within the Free-State and KwaZulu Natal provinces (surrounding the landlocked country of Lesotho) with higher-than-average CMI values across all population groups. This means that individuals in these districts are expected to incur higher-than-average healthcare utilization than what would be expected based on their age profile alone.

### Geographical distribution of claiming general practitioners and specialists

Inequities in access to healthcare in SA are largely due to the urban-rural divide and disproportionate concentration of GPs and SPs in private practice primarily in urban areas [25]. In order to contextualize the density of healthcare providers in relation to the density of the population in various districts, a comparison of the ratio of claiming general practitioners (GPs) and claiming medical and surgical specialists (SPs) per 1000 commercially insured study lives was performed. The provider density metric was calculated as the number of providers within a 5 km radius of an individual expressed as a proportion of the total population that have access to the same providers based on a 5 km radius.

**Fig. 3:**
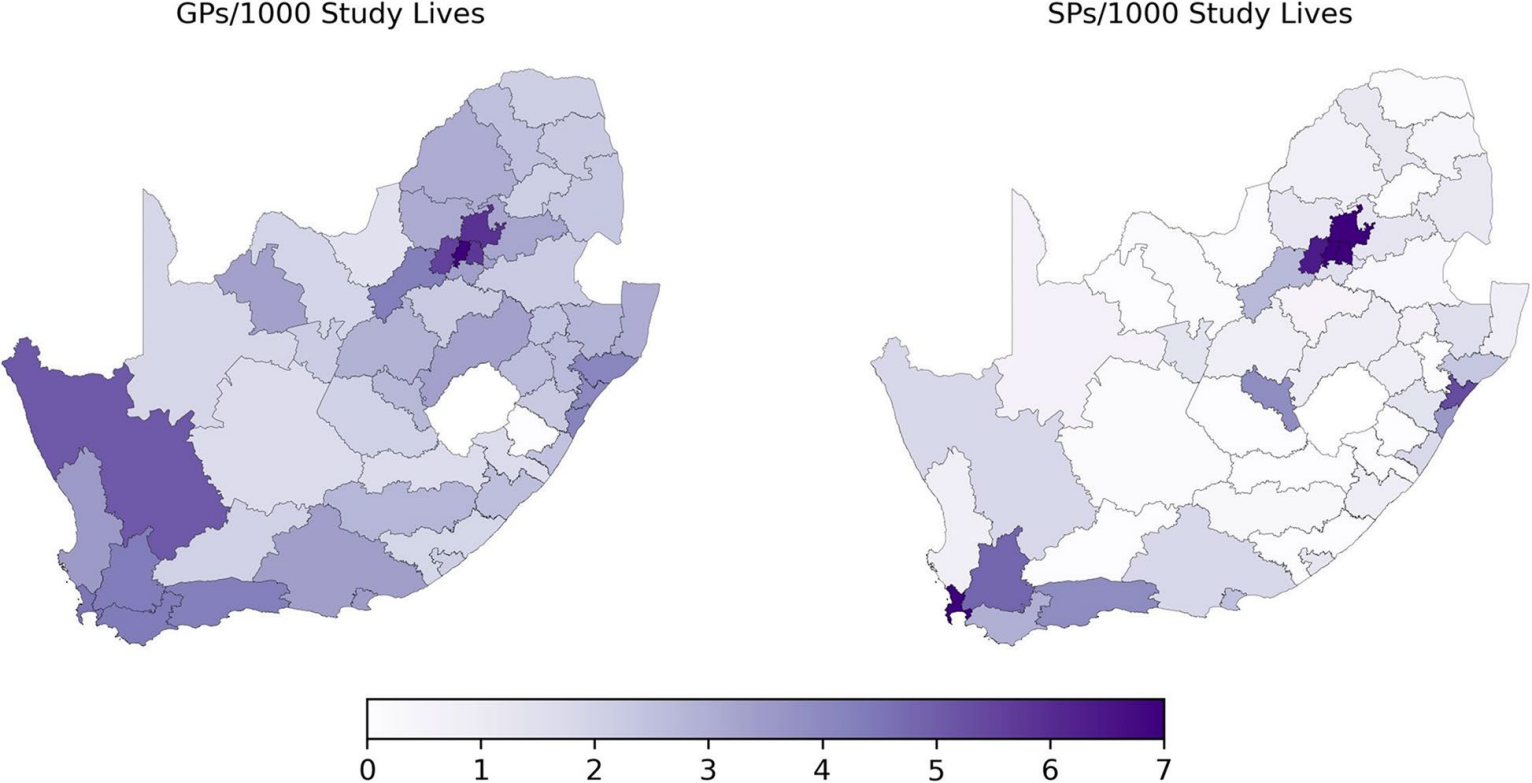
Geographical distribution of claiming general practitioners and specialists per 1000 commercially insured study lives per district. (Source: Authors’ work)

Based on data of claiming providers in 2016 and 2017 servicing the study population, higher numbers of GPs and SPs appear to be amongst the districts of the Gauteng and Western Cape Provinces which also have the highest number of lives and population density (Fig. 3).

Correlation coefficients were calculated to test the relationship between GP and SP density per 1000 study lives and by CMI. Based on the available data, a moderately weak correlation was found between the density of GPs (Pearson correlation coefficient = 0.37) and density of SPs (Pearson correlation coefficient = 0.51) in relation to population density. In contrast with these results, virtually no notable correlation was found between the density of GPs and SPs and disease burden (CMI) (Pearson correlation coefficients of -0.008 and 0.011 respectively). A strong positive correlation was found between the number of GPs/1000 and SPs/1000 study lives (Pearson correlation coefficient = 0.78) indicating that SPs tend to practice in areas with more GPs and vice versa.

**Fig. 4:**
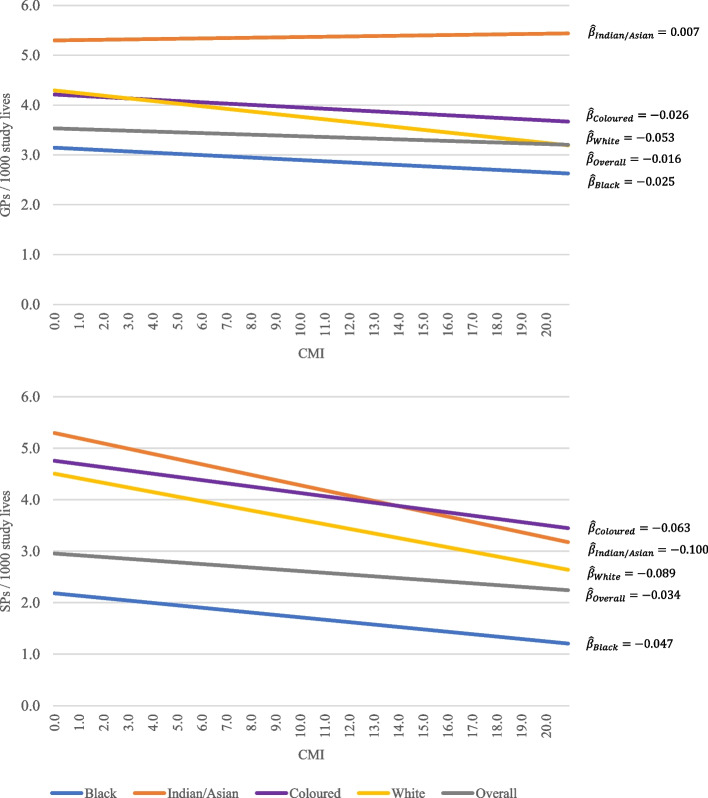
Relationship between morbidity (ACG risk scores) and general practitioner density (GPs per 1000 commercially insured lives) stratified by population group (top) and specialist density (SPs per 1000 commercially insured lives) stratified by population group (bottom). The intercepts and slopes of the lines were calculated by fitting a separate simple linear regression model between provider density and ACG risk scores for each population group. (Source: Authors’ work)

These results indicated a tendency for physicians to practice in areas with high numbers of lives (i.e., insurance enrollees) and not necessarily where disease burden may be highest. This was confirmed by our regression analysis which showed a negative relationship between GP density and ACG risk scores for the overall population and for three of the four major population groups, namely Black Africans, Whites, and Coloured (Fig. 4 top). Only the Indian/Asian population group showed a slightly positive relationship between GP density and CMI. Compared to the correlation of GP density and ACG risk scores, a stronger negative correlation was found between ACG risks scores and SP density across all population groups (Fig. 4 bottom).

Table 1 presents the results from the GP and SP density regression models. The regression analysis confirms that population density is the strongest predictor of high physician density. While this is true for GPs and SPs, the relationship is more than twice as strong with respect to SPs than to GPs ($\widehat{\beta }$=10.81 (*p* < 0.001), partial *R*^2^ = 0.2668 vs $\widehat{\beta }$=4.58 (*p* < 0.001), partial *R*^2^ = 0.1377)). Additionally, results revealed a positive relationship between practitioner density and age (e.g., $\widehat{\beta }$=0.34 (*p *< 0.001), partial *R*^2^ = 0.0004 for SPs). Table 1 also shows that the Black African population group was found to, on average, reside in areas with lower GP and SP density after adjusting for other relevant factors ($\widehat{\beta }$=-0.19 (*p* < 0.001) for GPs and $\widehat{\beta }$=-0.43 (*p* < 0.001) for SPs). Conversely, the Indian/Asian population was found to live in areas with the highest GP and SP density on average ($\widehat{\beta }$=1.92 (*p* < 0.001) for GPs and $\widehat{\beta }$=2.51 (*p* < 0.001) for SPs).

**Table 1:** GP and SP distribution model variables and specifications (Source: Authors’ work)

	$\widehat{\beta }$	S.E	95% CI	*p*-value	Partial *R*^2^
GP Model Variables					
Intercept	2.6046	0.0100	(2.5850, 2.6250)	< 0.001	
Age adjusted ACG risk score	-0.9575	0.0470	(-1.0490, -0.8660)	< 0.001	0.0002
Age	0.1942	0.0070	(0.1800, 0.2080)	< 0.001	0.0003
Female	-0.0057	0.0030	(-0.0120, 0.0001)	0.0540	0.0000
Black	-0.1969	0.0100	(-0.2160, -0.1770)	< 0.001	0.0504
White	0.7650	0.0100	(0.7450, 0.7850)	< 0.001	
Coloured	0.7928	0.0110	(0.7710, 0.8140)	< 0.001	
Indian/Asian	1.9249	0.0120	(1.9010, 1.9490)	< 0.001	
5 km Neighbour density	4.5802	0.0070	(4.5660, 4.5940)	< 0.001	0.1377
*R*^*2*^ = *0.184, adjusted R*^*2*^ = *0.184*					
SP Model Variables					
Intercept	0.9000	0.0160	(0.8690, 0.9310)	< 0.001	
Age adjusted ACG risk score	-1.8091	0.0730	(-1.9520, -1.6660)	< 0.001	0.0002
Age	0.3487	0.0110	(0.3270, 0.3700)	< 0.001	0.0004
Female	-0.0046	0.0050	(-0.0140, 0.0040)	0.3190	0.0000
Black	-0.4303	0.0150	(-0.4610, -0.4000)	< 0.001	0.0676
White	1.4967	0.0160	(1.4650, 1.5280)	< 0.001	
Coloured	1.9480	0.0170	(1.9140, 1.9820)	< 0.001	
Indian/Asian	2.5111	0.0190	(2.4740, 2.5490)	< 0.001	
5 km Neighbour density	10.8092	0.0110	(10.7870, 10.8310)	< 0.001	0.2668
*R*^*2*^ = *0.315, adjusted R*^*2*^ = *0.315*					

## Discussion

Disparities in access to healthcare resources is a major barrier to equitable health. Prior research in measuring inequities of healthcare resources have often used single disease outcomes to control for underlying burden of diseases. Indeed, leveraging population-level comorbidity measures for health disparity research has been limited. To address this gap, this study compares the geographical distribution of comorbidity and its associated healthcare utilization implications among commercially insured individuals in South Africa (SA) relative to the distribution of general practitioners (GPs) and specialist physicians (SPs).

Our results showed inequalities in the distribution of GPs and SPs from whom our 2.6 M study lives claimed treatment in terms of geography, population group, and morbidity. We found that the distribution of practitioners was not only correlated with population size in absolute terms (which is desired), but also with population density. This implies that individuals in more densely populated metropoles generally have access to more GPs and SPs per capita compared to those in rural areas. The relationship between practitioner density and age could include an association between age and wealth and hence residence in more affluent neighborhoods, and self-selection with older individuals choosing to reside close to health centers. The Indian/Asian and White population groups had on average highest CMIs with the Indian/Asian population geographically concentrated in areas exhibiting higher numbers of GPs and SPs. The Black African population group had, on average, access to fewer GPs and SPs per capita than all other population groups, before and after adjusting for differences in age, gender, ACG risk scores and population density. Overall, we found no positive correlation between total disease burden as measured by ACG risk scores, and GP and SP density.

Legacy inequities in access to healthcare in SA as a remnant of apartheid and the current urban–rural divide are not self-correcting and will rely on current and future policymakers to remedy them through social and political policies [30–32]. Classifying districts by CMI offers a unique perspective of geographical areas expecting to incur highest and lowest healthcare utilization whether due to real increased or lower healthcare needs and even potentially unmet needs which could aid resource allocation and service planning [6, 21]. Nonetheless, CMI captured in insurance claims may underrepresent the actual total burden of diseases in geographical areas with poor access to healthcare resources (i.e., complete data not captured due to lower healthcare encounters). As such, the misallocation of resources relative to morbidity as measured in our paper may be understated. Additionally, differences in healthcare seeking behavior may also explain different utilization patterns among individuals of the same disease burden [33].

Based on the study results, the distribution of GPs and SPs in the private healthcare sector may be disproportionately influenced by factors such as economic opportunity and the social desires of healthcare practitioners compared to a broader view of national population needs. While a concentration of physicians in highly populated areas and the co-location of GPs and SPs may be expected and therefore unsurprising, it reaffirms the need for a comprehensive approach, targeting factors such as the economic upliftment of regions in need and improvement in career development opportunities, to provide attractive opportunities to healthcare professionals to move and practice in such geographies [34, 35]. Health policy approaches may include direct incentives for both the public sector and the private health plans to improve primary healthcare coverage in areas where disease burden may be inadequately resourced [34–37].

While the equitable distribution of healthcare resources is desired, its practical realization may be hindered by existing national shortages of healthcare professionals in SA [38], and where limited specialized healthcare services exist. Such redistribution efforts typically require sharing of expensive hospital resources such as hospital beds, operating theatres and other medical equipment as is needed for tertiary care [38].

In this study, we have presented a novel approach to measure inequity by considering the total burden of disease at a population level. This analysis can be performed regularly with minimal cost using insurance claims data that are routinely collected across all healthcare providers. Using this approach on a centralized database, where information from public and private entities are pooled, could be used for population-level disparities analysis while considering the burden of comorbidities. Furthermore, with the introduction of a national electronic health record (EHR) system in SA, such information can also be collected and acted upon in near real-time.

### Limitations

The results should be interpreted within the boundaries of the following limitations: First, the study population were mostly employed and are likely to be healthier due to better socio-economic circumstances in general. Hence, our study may understate results especially in geographical areas impacted by high unemployment rates [27] and unmet healthcare needs [39]. Postal codes recorded by the health plan administrator were considered a reliable data source of the latest address for individuals and healthcare providers included in this study. Missing postal codes or having multiple address locations recorded may have resulted in unrepresented individuals causing some geographical bias in the study population [40]. This study was based on a sample of commercially insured individuals of specific client health plans to a large claims administrator in SA. The distribution of members across these health plans means that some districts (such as the City of Johannesburg in Gauteng) were more poorly represented than others.

Second, to protect the anonymity of the members and health plans included in this study, it was not possible to assess associations between specific benefit package designs and healthcare utilization (i.e., CMI) without potentially identifying health plans through benefits offered and consequently individuals. However, the legislated implementation of a PMB package in SA ensures a standard minimum level of treatment, which health plans in the private sector are obligated to fund. Thus, variability of health benefit packages was assumed to have less impact on the disparity associated with affordability and access to healthcare.

Third, the counts of healthcare practitioners used in this study are limited to the claiming providers having treated the study individuals over 2016 and 2017 and do not represent the total number of physicians in SA. The correlations involving healthcare provider density and number of lives or CMI portrayed in this study were performed to test whether relationships between these factors may be detected using the available dataset. It is acknowledged that healthcare providers may service more than one district and may treat commercially and non-commercially insured patients in both the public and private sectors. To minimize overstating the paucity of healthcare practitioners, our methodology comprised of a radius within which to measure individual and healthcare provider density. Data on the total number of SA healthcare practitioners in various geographical locations and in the private and public sectors was not readily available from public resources for use in our study. Considering the paucity of access to such provider-level information in SA, which is important for planning purposes, our approach could be considered a practical method to leverage readily available data to count healthcare practitioners for a population of insured patients.

Fourth, the centroid method to measure provider density (number of providers per 1000 individuals) overstates access in larger wards typically found outside the urban metropoles and more rural areas. Given the availability of postal code data for individuals and providers available in our study (and not exact addresses for confidentiality purposes), measurement of actual access to providers was not possible. While this is a limitation of the study, since most of the study population (72%) live in wards classified as urban by Statistics South Africa, the inequities studied in this paper are therefore less impacted by this limitation and could be considered primarily of urban areas rather than between urban and rural areas. Our results show that despite provider density likely overstated in rural areas, these areas are still underserved relative to their disease burden.

Fifth and finally, we conducted stratified linear regressions to measure the difference of correlation between GP/SP density and CMI across population subgroups. This study did not intend to analyze the effect of geography on CMI, partially due to the study population not representing a balanced sample of all SA populations. Furthermore, such analysis will be prone to potential unmeasured moderators across geographic groupings. Hence future research on geographical disparities of GP/SP density versus CMI should apply a multi-level modeling technique to control for potential ecological variables not measured by individual-level insurance claims data.

## Conclusions

Our study assessed the distribution of comorbidities among 2.6 million commercially insured individuals in SA as well as the distribution of treating GPs and SPs by district using claims data. Results of our analysis showed a greater tendency for GPs and SPs to be geographically located in areas accounting for the most individuals, suggesting an opportunity to improve distribution of resources in areas exhibiting greater disease burden with lower access to GP and SP care. While this finding may be unsurprising and even expected, this study suggests that measuring morbidity using claims data or data that describes healthcare services being provided, could be a practical way to obtain important insights to morbidity surveillance and healthcare services planning that would otherwise require more expensive, time-consuming studies for which the results would inevitably be delayed past the point policy decisions may need to be taken.

The use of a measure that considers the total burden of disease (i.e., acute, and chronic conditions contributing to current and future healthcare costs) presents CMI as a novel approach to explore disparities and inequities in health by providing a means of detecting disparities in utilization of healthcare resources that contradict the level of morbidity identified from age, gender and diagnosis information (i.e., detecting utilization not at the expected level of demographics and diagnoses). The disparities in resources compared to observed comorbidities have implications on individual population groups requiring further study.

This study linked the geographical distribution of comorbidity (i.e., disease burden) and the distribution of healthcare resources using the same measure (i.e., CMI) applied to routinely collected data which could provide vital information towards the more equitable distribution of resources and bundling of healthcare services to meet the specific healthcare needs of communities and improve overall population health. Conceptually, the use of records of health services obtained by patients in the public sector could substitute the role played by claims for services rendered which makes the use of such a measure for timely decision-making both practical and scalable.

## Supplementary Information

**Additional file 1. **Study population characteristics. A table comparing the study population to the 2011 South African Census population.

**Additional file 2.** District and provincial boundaries, South Africa. Labelled map that illustrates the district boundaries in South Africa within each province.

**Additional file 3. **Regression sensitivity analysis. Figures illustrating the analysis performed to test the sensitivity of the regression results to the 5km radius versus a 10km radius distance, as well as the suitability of linear regression compared to other regression methods.

## Data Availability

The data that support the findings of this study are available from Medscheme Pty (Ltd) South Africa, but restrictions apply to the availability of these data, which were used under license for the current study, and so are not publicly available. Data are, however, available from the authors upon reasonable request and with permission of Medscheme Pty (Ltd) South Africa.

## Abbreviations

SA: South Africa
ACG®: Adjusted Clinical Grouper
CMI: Comorbidity index
PMBs: Prescribed Minimum Benefits
GPs: General practitioners
SPs: Specialist physicians
