# Supplementary material for: Measuring the geographic disparity of comorbidity in commercially insured individuals compared to the distribution of physicians in South Africa
Source: BMC Prim Care. 2022 Nov 17;23:286. doi: 10.1186/s12875-022-01899-1 (PMC9673280; doi:10.1186/s12875-022-01899-1)
Supplement: Supplementary file 1 — Additional file 1. Study population characteristics. A table comparing the study population to the 2011 South African Census population. [file 12875_2022_1899_MOESM1_ESM.docx]

**Additional file 1: Study population characteristics**

Table A1.Characteristics of the study population compared to 2011 South African Census population

|  | **Study Population 2016 2017** | **South Africa Census 2011** |
| --- | --- | --- |
| **Unique Individuals** | 2,638,955 | 51,764,899 |
| Wards | 1427 | 4277 |
| Districts | 52 | 52 |
| Provinces | 9 | 9 |
| **Sex** |  |  |
| Female | 1,459,269 (55.3%) | 26,579,527 (51.3%) |
| **Population Groups** |  |  |
| Black African | 1,743,515 (66.1%) | 40,996,454 (79.2%) |
| Indian/Asian | 102,229 (3.9%) | 1,286,789 (2.5%) |
| Coloured | 203,298 (7.7%) | 4,614,896 (8.9%) |
| White | 530,274 (20.1%) | 4,586,336 (8.9%) |
| Unknown | 59,639 (2.3%) | 280,423 (0.5%) |
| **Age Bands** |  |  |
| 00-04 | 251,838 (9.5%) | 5,684,973 (11.0%) |
| 05-09 | 277,237 (10.5%) | 4,819,353 (9.3%) |
| 10-14 | 257,808 (9.8%) | 4,594,492 (8.9%) |
| 15-19 | 245,690 (9.3%) | 5,003,087 (9.7%) |
| 20-24 | 94,063 (3.6%) | 5,374,063 (10.4%) |
| 25-29 | 112,642 (4.3%) | 5,058,738 (9.8%) |
| 30-34 | 180,821 (6.9%) | 4,028,532 (7.8%) |
| 35-39 | 186,664 (7.1%) | 3,467,343 (6.7%) |
| 40-44 | 205,362 (7.8%) | 2,948,218 (5.7%) |
| 45-49 | 209,260 (7.9%) | 2,619,908 (5.1%) |
| 50-54 | 192,386 (7.3%) | 2,217,920 (4.3%) |
| 55-59 | 152,690 (5.8%) | 1,797,131 (3.5%) |
| 60-64 | 99,006 (3.8%) | 1,385,535 (2.7%) |
| 65-69 | 66,348 (2.5%) | 957,668 (1.9%) |
| 70-74 | 46,011 (1.7%) | 748,204 (1.4%) |
| 75-79 | 30,855 (1.2%) | 481,216 (0.9%) |
| 80-84 | 17,575 (0.7%) | 322,870 (0.6%) |
| 85+ | 12,699 (0.5%) | 255,648 (0.5%) |
| **Province** |  |  |
| Eastern Cape | 292,154 (11.1%) | 6,560,024 (12.7%) |
| Free State | 221,538 (8.4%) | 2,745,290 (5.3%) |
| Gauteng | 557,680 (21.1%) | 12,271,736 (23.7%) |
| KwaZulu-Natal | 458,618 (17.4%) | 10,266,802 (19.8%) |
| Limpopo | 260,984 (9.9%) | 5,404,032 (10.4%) |
| Mpumalanga | 252,359 (9.6%) | 4,039,488 (7.8%) |
| North West | 194,919 (7.4%) | 3,509,672 (6.8%) |
| Northern Cape | 83,159 (3.2%) | 1,145,529 (2.2%) |
| Western Cape | 317,544 (12.0%) | 5,822,326 (11.2%) |
| **Exposure** |  |  |
| Average membership months per year | 11.8 | - |
| **Morbidity status** |  |  |
| ACG risk score / Comorbidity index (CMI) | 0.998 | - |
