# Supplementary material for: Measuring the geographic disparity of comorbidity in commercially insured individuals compared to the distribution of physicians in South Africa
Source: BMC Prim Care. 2022 Nov 17;23:286. doi: 10.1186/s12875-022-01899-1 (PMC9673280; doi:10.1186/s12875-022-01899-1)
Supplement: Supplementary file 2 — Additional file 2. District and provincial boundaries, South Africa. Labelled map that illustrates the district boundaries in South Africa within each province. [file 12875_2022_1899_MOESM2_ESM.docx]

**Additional file 2: District and provincial boundaries, South Africa**


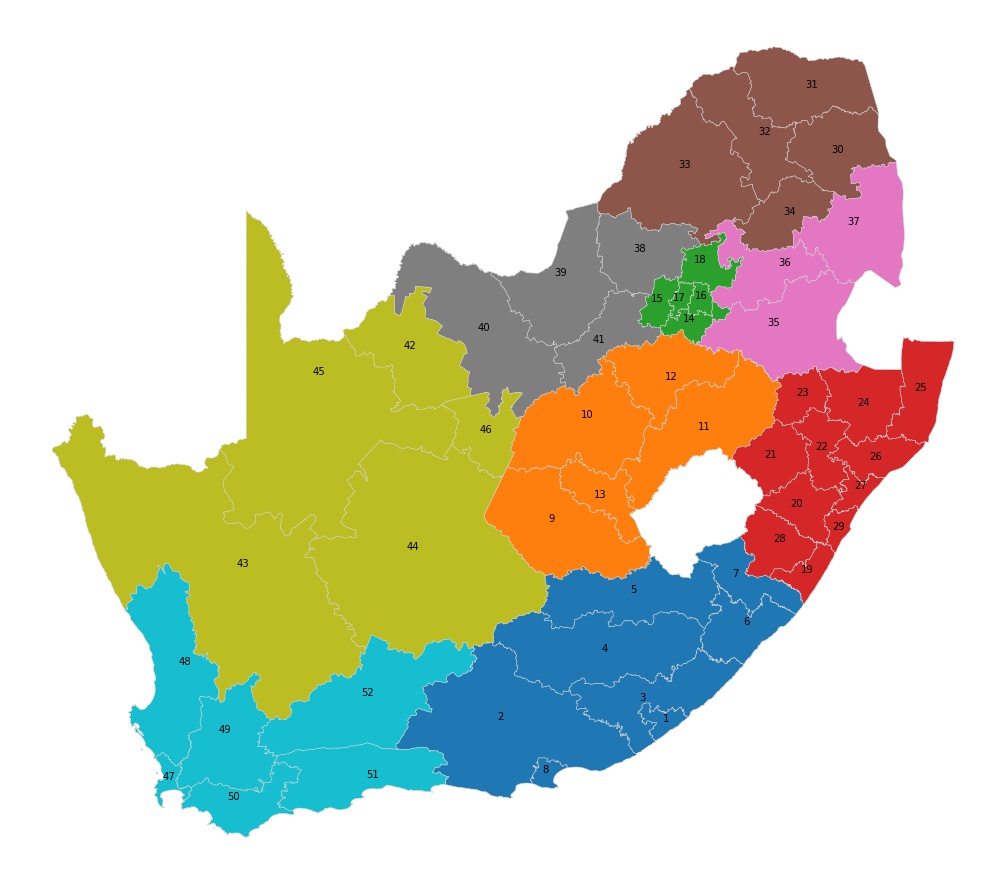


Figure B1. Map of South Africa illustrating the district boundaries for all 9 provinces

|  | ***Eastern Cape*** |  | ***KwaZulu-Natal*** |  | 37 | Ehlanzeni |
| --- | --- | --- | --- | --- | --- | --- |
| 1 | Buffalo City | 19 | Ugu |  |  | ***North West*** |
| 2 | Cacadu | 20 | UMgungundlovu |  | 38 | Bojanala |
| 3 | Amathole | 21 | Uthukela |  | 39 | Ngaka Modiri Molema |
| 4 | Chris Hani | 22 | Umzinyathi |  | 40 | Dr Ruth Segomotsi Mompati |
| 5 | Joe Gqabi | 23 | Amajuba |  | 41 | Dr Kenneth Kaunda |
| 6 | O.R.Tambo | 24 | Zululand |  |  | ***Northern Cape*** |
| 7 | Alfred Nzo | 25 | Umkhanyakude |  | 42 | John Taolo Gaetsewe |
| 8 | Nelson Mandela Bay | 26 | Uthungulu |  | 43 | Namakwa |
|  | ***Free State*** | 27 | iLembe |  | 44 | Pixley ka Seme |
| 9 | Xhariep | 28 | Sisonke |  | 45 | Siyanda |
| 10 | Lejweleputswa | 29 | eThekwini |  | 46 | Frances Baard |
| 11 | Thabo Mofutsanyane |  | ***Limpopo*** |  |  | ***Western Cape*** |
| 12 | Fezile Dabi | 30 | Mopani |  | 47 | City of Cape Town |
| 13 | Mangaung | 31 | Vhembe |  | 48 | West Coast |
|  | ***Gauteng*** | 32 | Capricorn |  | 49 | Cape Winelands |
| 14 | Sedibeng | 33 | Waterberg |  | 50 | Overberg |
| 15 | West Rand | 34 | Greater Sekhukhune |  | 51 | Eden |
| 16 | Ekurhuleni |  | Mpumalanga |  | 52 | Central Karoo |
| 17 | City of Johannesburg | 35 | Gert Sibande |  |  |  |
| 18 | City of Tshwane | 36 | Nkangala |  |  |  |
