# Supplementary material for: Measuring the geographic disparity of comorbidity in commercially insured individuals compared to the distribution of physicians in South Africa
Source: BMC Prim Care. 2022 Nov 17;23:286. doi: 10.1186/s12875-022-01899-1 (PMC9673280; doi:10.1186/s12875-022-01899-1)
Supplement: Supplementary file 3 — Additional file 3. Regression sensitivity analysis. Figures illustrating the analysis performed to test the sensitivity of the regression results to the 5km radius versus a 10km radius distance, as well as the suitability of linear regression compared to other regression methods. [file 12875_2022_1899_MOESM3_ESM.docx]

**Additional file 3: Regression sensitivity analysis**

*Sensitivity of results to the 5km radius*

In our analysis, reasonable access to general practitioners (GPs) and specialists (SPs) was defined as being within a 5km radius of a provider. The choice of 5km as the threshold was based on guidance from the Department of Health, and is based on a maximum walking time of one hour at a normal pace (approximately 4.5km/hour).

The exact locations of the commercially insured individuals and physicians in our study were not available in our data to protect the identity of study participants. As a result, access to the electoral wards in which the study individuals resided and providers practiced which was provided, was used rather than exact locations. While most study individuals resided in electoral wards where the distance between the center of the ward to the center of the nearest neighboring ward is less than the 5km radius, we also performed the regression analysis using a 10km radius to test the sensitivity of the choice of radius to our results.

Figure C1 shows a comparison of the regression coefficients (scaled to values between 0 and 1 for comparative purposes) produced for the GP density model using 5km and 10km access thresholds respectively. We conclude that the same findings would have been reached had a 10km radius been used in our study.

Figure C1. Comparison of the regression coefficients for the GP density model using a 5km and 10km radius to calculate provider density.

*Suitability of linear regression*

As is common with healthcare datasets, our response variables (GP and SP density) are right skewed (see Figure C2 below) and thus violates the normality assumption.


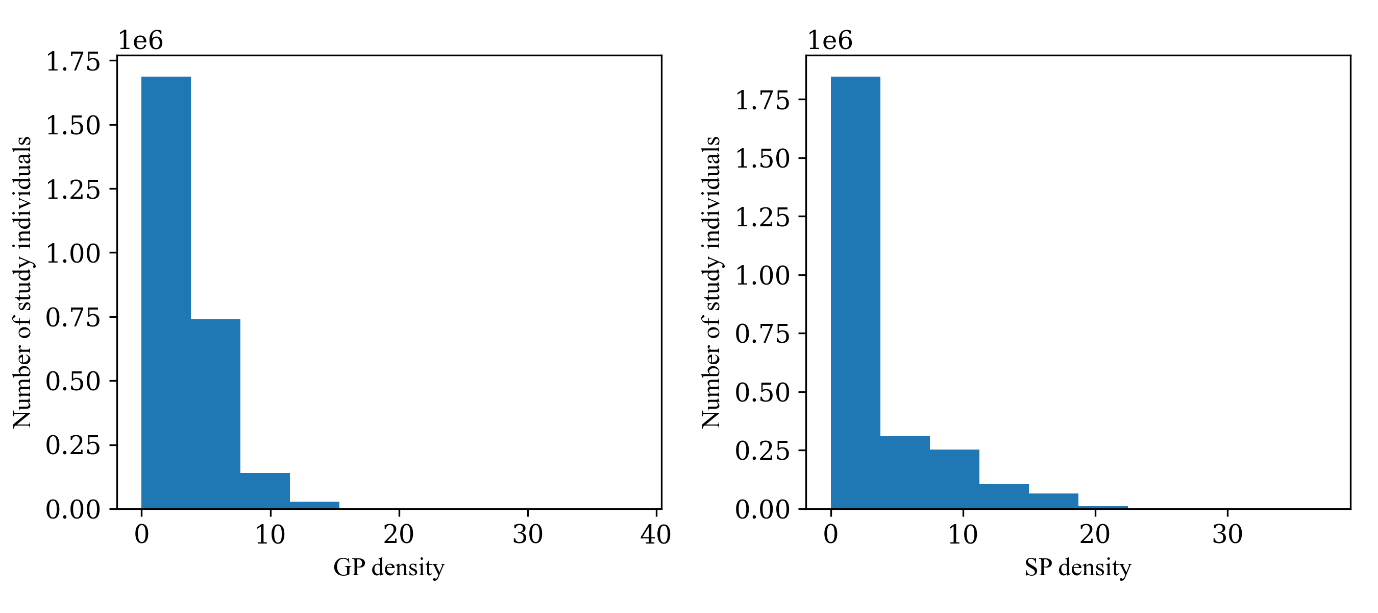


Figure C2. Histograms showing the distribution of GP (left) and SP (right) density

For this analysis, linear regression was considered valid given the large sample size^[[1]](#footnote-2)^, and that transformations of the response variable to meet the normality assumption would introduce other biases. To measure the robustness of our regression point estimates, other model types were tested, namely:

- Linear regression on a log transformed response variable.
- Logistic regression on a binary formulation where:
  - 1 = High GP/SP density (density above the median); 0 = Other
  - 0 = High GP/SP density (density above the 75th percentile); 0 = Other

Directionally, all models lead to the same conclusions. For example, Figure C3 below compares the regression coefficients (scaled to values between 0 and 1 for comparative purposes) of the model used in this study (identified as GP-5km-OLS) to the other models tested with GP density as the response variable.

Figure C3. Comparison of the regression coefficients for the GP density model for different regression methods and model specifications

1. Lumley, T., Diehr, P., Emerson, S., & Chen, L. (2002). The importance of the normality assumption in large public health data sets. In *Annual Review of Public Health* (Vol. 23, pp. 151–169). <https://doi.org/10.1146/annurev.publhealth.23.100901.140546>

   Schmidt, A. F., & Finan, C. (2018). Linear regression and the normality assumption. In *Journal of Clinical Epidemiology* (Vol. 98, pp. 146–151). https://doi.org/10.1016/j.jclinepi.2017.12.006 [↑](#footnote-ref-2)
